# NLRP3 Inflammasome Participates in Host Response to *Neospora caninum* Infection

**DOI:** 10.3389/fimmu.2018.01791

**Published:** 2018-07-30

**Authors:** Xiaocen Wang, Pengtao Gong, Xu Zhang, Shan Li, Xiangyun Lu, Chunyan Zhao, Qile Yu, Zhengkai Wei, Yongjun Yang, Qun Liu, Zhengtao Yang, Jianhua Li, Xichen Zhang

**Affiliations:** ^1^College of Veterinary Medicine, Jilin University, Changchun, Jilin, China; ^2^National Animal Protozoa Laboratory, Key Laboratory of Animal Epidemiology of the Ministry of Agriculture, College of Veterinary Medicine, China Agricultural University, Beijing, China

**Keywords:** *Neospora caninum*, NLRP3 inflammasome, IL-18, IFN-γ, host defense

## Abstract

*Neospora caninum* is an intracellular protozoan parasite closely related to *Toxoplasma gondii* that mainly infects canids as the definitive host and cattle as the intermediate host, resulting in abortion in cattle and leading to financial losses worldwide. Commercial vaccines or drugs are not available for the prevention and treatment of bovine neosporosis. Knowledge about the hallmarks of the immune response to this infection could form the basis of important prevention strategies. The innate immune system first responds to invading parasite and subsequently initiates the appropriate adaptive immune response against this parasite. Upon infection, activation of host pattern-recognition receptors expressed by immune cells triggers the innate immune response. Toll-like receptors, NOD-like receptors, and C-type lectin receptors play key roles in recognizing protozoan parasite. Therefore, we aimed to explore the role of the NLRP3 inflammasome during the acute period of *N. caninum* infection. *In vitro* results showed that *N. caninum* infection of murine bone marrow-derived macrophages activated the NLRP3 inflammasome, accompanied by the release of IL-1β and IL-18, cleavage of caspase-1, and induction of cell death. K^+^ efflux induced by *N. caninum* infection participated in the activation of the inflammasome. Infection of mice deficient in NLRP3, ASC, and caspase-1/11 resulted in decreased production of IL-18 and reduced IFN-γ in serum. Elevated numbers of monocytes/macrophages and neutrophils were found at the initial infection site, but they failed to limit *N. caninum* replication. These findings suggest that the NLRP3 inflammasome is involved in the host response to *N. caninum* infection at the acute stage and plays an important role in limiting parasite growth, and it may enhance Th1 response by inducing production of IFN-γ. These findings may help devise protocols for controlling neosporosis.

## Introduction

*Neospora caninum* is an apicomplexan parasite that infects a broad range of warm-blooded animals and leads to neosporosis worldwide (1–3). *N. caninum* usually progresses through three stages: tachyzoite, bradyzoite, and sporozoite. In intermediate host, fast dissemination of replicating tachyzoites is responsible for the acute phase of neosporosis (4). Neosporosis often causes abortion especially in dairy cattle and leads to global economic losses (5); however, there is no treatment or vaccine available against this disease in cattle (6). Increasing attention has been paid to defining the role of the host immune response in *N. caninum* infection and to exploring the immune functions that influence control of infection and disease development (7). Host immune response against *N. caninum* infection is an essential determinant of neosporosis (1, 6, 8). Similar to *Toxoplasma gondii, N. caninum* induces a Th1-type protective immune response mainly mediated by the production of the proinflammatory cytokines IFN-γ and IL-12p40 (6, 9). The host innate immune system plays an important role in recognizing microbes and shaping an appropriate adaptive immune response during the early stage of the infection (10). *N. caninum* can activate different pattern-recognition receptors (PRRs) of innate immune cells after infecting the host, such as TLR2 (11), TLR3 (12), TLR11 (13, 14), NOD2 (10), Dectin-1 (9), and NLRP3 (15), and these PRRs consequently induce the host immune response against this parasite.

NLRP3 is a sensor protein of the NLR family and localizes to the cytoplasm. When it senses various pathogen-associated molecular patterns (PAMPs) or danger-associated molecular patterns (DAMPs), NLRP3 interacts with the pyrin domain of ASC, and then the CARD domain of ASC binds the CARD domain of caspase-1 to form the NLRP3 inflammasome through protein–protein domain interactions (16). The assembly of the NLRP3 inflammasome leads to caspase-1 activation, and active caspase-1 cleaves pro-IL-1β/pro-IL-18 into their active forms, mediating the release of IL-1β and IL-18. On the other hand, active caspase-1 triggers an inflammatory form of cell death called pyroptosis by cleaving gasdermin D (GSDMD) into its N-terminal form which binds to the cell membrane and oligomerizes to form pores, leading to the rupture of cells (17).

Appropriate activation of the NLRP3 inflammasome facilitates the restriction of microbe growth (18, 19). In *T. gondii* infection, activation of the NLRP3 inflammasome and inflammasome-mediated IL-18 production play crucial roles in controlling the parasite and enhancing the host defense against *T. gondii* (20). *T. gondii* induces pyroptosis in rat macrophages by activating the NLRP1 inflammasome, which facilitates the killing of *T. gondii* (21). In addition, IL-1β and IL-18 are IL-1 family members, could contribute to the development of T cell responses, and shape adaptive immune response during infection (22). These cytokines are required to differentiate naïve T cells into effector forms, which include Th1, Th2, and Th17. In general, IL-1β is considered to support Th17 differentiation and promote IL-17 secretion during host response against extracellular pathogens (23). Whereas IL-18, serves as an inducer of IFN-γ production by Th1 cells, mainly promotes Th1 differentiation in synergy with IL-12 (24). In *Streptococcus pneumoniae* infection, pneumolysin can induce the secretion of IL-17A and IFN-γ *via* NLRP3 inflammasome signaling. Both IL-17A and IFN-γ are essential to protective immune response against pneumococcal infection (25). And various studies show that inflammasome-mediated IL-18 or IL-1β plays important roles in controlling pathogens and promoting adaptive immune response against infection (23, 24). Our previous study showed that NLRP3 inflammasome can be activated by *N. caninum* infection *in vitro*, but how the NLRP3 inflammasome mediates the immune response to *N. caninum* infection is unknown. In this study, we focus on exploring the role of the NLRP3 inflammasome in the *N. caninum*-induced immune response in the acute phase of infection.

## Materials and Methods

### Animals

Female C57BL/6 mice (5–8 weeks old) were obtained from the Laboratory Animal Center of Jilin University (Changchun, China). *Nlrp3^−/−^* mice were purchased from The Jackson Laboratory (Bar Harbor, ME, USA). *Asc^−/−^* and *Caspase-1/11^−/−^* mice were kindly provided by Dr. Feng Shao (National Institute of Biological Sciences, Beijing, China). The mice were maintained in isolator cages, with sterile food and water in the animal house of the Laboratory Animal Center of Jilin University. All animal experiments were performed in strict accordance with guidelines from the Animal Welfare and Research Ethics Committee under protocols approved by Jilin University.

### Parasites and Experimental Infection

Tachyzoites from *N. caninum* (Nc-1) parasites were used for all studies. Parasites were maintained by serial passages in Vero cells, which were cultured in RPMI-1640 medium (Life Technologies, Grand Island, NY, USA) supplemented with 2 mM glutamine, 1 mM sodium pyruvate, 10 mM HEPES, 100 U/ml penicillin, 100 µg/ml streptomycin, and 2% heat-inactivated fetal bovine serum (FBS; BI, Shanghai, China). Parasites were harvested and collected by centrifuging at 1,500 × *g* for 30 min with a 40% Percoll (GE Healthcare, Uppsala, Sweden) solution (v/v) to remove host cell debris. The parasite suspension was collected, washed twice with RPMI-1640 and centrifuged at 1,000 × *g* for 10 min; then the *N. caninum* concentration was determined in a hemocytometer. In animal experiments, mice (5–8 weeks old) were infected intraperitoneally with either 1 × 10^7^ or 2 × 10^7^ Nc-1 tachyzoites diluted in 500 µl phosphate-buffered saline.

### Cell Culture and Stimulation

Bone marrow-derived macrophages (BMDMs) were generated from bone marrow stem cells of wild-type (WT), *Nlrp3^−/−^, Asc^−/−^*, and *Caspase-1/11^−/−^* mice. Briefly, stem cells were cultured on 10-cm diameter polystyrene plates for 7 days in RPMI-1640 medium, containing 2 mM glutamine, 1 mM sodium pyruvate, 10 mM HEPES, 100 U/ml penicillin, 100 µg/ml streptomycin, 10% heat-inactivated FBS, and 25% cell-conditioned medium that was obtained from the supernatant of confluent L929 cells.

Cells were counted and seeded in 6-well plates at 1.5 × 10^6^ cells/well. For experiments, the medium was changed to complete medium (RPMI) plus 1% FBS. In the experimental group, BMDMs were pre-treated with LPS (100 ng/ml) for 2 h, then washed twice with PBS, and infected with *N. caninum* at various multiplicities of infection (MOI = 1:1 or 3:1; parasite:cell) for the indicated times. BMDMs treated with medium alone were used as negative controls, and BMDMs pre-treated with LPS and then stimulated by ATP (5 mM, 30 min; Sigma, Shanghai, China) were used as positive controls. Cell viability was assessed at 3 h post infection (p.i.) by using a TransDetect Cell Counting Kit (Trans, Beijing, China). Culture supernatants were removed at various time points and immediately stored at −20°C for cytokine measurements. In select experiments, supernatants were taken for the lactate dehydrogenase assay (Roche Diagnostics, Mannheim, Germany) according to the manufacturer’s protocol, as a measure of cell death.

To monitor whether K^+^ efflux contributed to the inflammasome activation in *N. caninum*-infected BMDMs, BMDMs were treated with 50 µM glyburide (an inhibitor of the NLRP3 inflammasome by inhibiting K^+^ efflux; Selleck, Shanghai, China) for 60 min before stimulation, and the BMDMs were then stimulated with LPS priming plus *N. caninum* for 3 h at an MOI of 3:1 (parasite:cell). BMDMs cultured with 0.05% DMSO were used as a negative control.

### Western Blot

Precipitated supernatants and BMDMs extracts were analyzed by immunoblot as described previously (15). The primary antibodies used were anti-mouse IL-1β (AF-401, R&D, Minneapolis, MN, USA), anti-mouse caspase-1 (p20) (AG-20B-0042, Adipogen, Liestal, Switzerland), anti-ASC (AG-25B-0006-C100, Adipogen, Liestal, Switzerland), anti-NLRP3 (AG-20B-0014, Adipogen, Liestal, Switzerland), and anti-mouse β-actin (Proteintech, Wuhan, China).

### Determination of Parasite Burden by Real-Time Quantitative PCR (qPCR)

Bone marrow-derived macrophages were infected with Nc-1 tachyzoites (MOI = 1) and collected at 24 h p.i. For *in vivo* assays, mice were intraperitoneally infected with 1 × 10^7^ Nc-1 tachyzoites, and the peritoneal cells were extracted at 7 days p.i. for analysis of parasite burden at the initial infection site. Fragments of brain, lung, and heart were collected at day 5 p.i. (2 × 10^7^ group). All samples were stored at −20°C.

The parasite replication in cells and tissues was monitored as previously described (26) by performing a qPCR analysis of the parasite DNA. Genomic DNA from 8 × 10^7^ Nc-1 tachyzoites and total DNA from infected cells and tissues were extracted using a Genomic DNA Extraction Kit (TIANGEN, Beijing, China) according to the manufacturer’s protocol. The total DNA (500 ng) from samples was used as a template in qPCR analyses with FastStart Universal SYBR Green Master. A primer pair specific for the Nc5 sequence of *N. caninum* (Forward: 5’-ACTGGAGGCACGCTGAACAC-3’, Reverse: 5’- AACAATGCTTCGCAAGAGGAA-3’) was used to amplify a 76-bp DNA fragment. The parasite number was determined by a standard curve performed with DNA isolated from *N. caninum* tachyzoites, ranging from 3.2 to 3.2 × 10^5^ parasites, included in each run.

### ELISA

Supernatants from cell culture, peritoneal lavage fluid, and serum from mice were measured by mouse IL-1β, IL-18, IFN-γ, IL-12, IL-10, IL-6, or TNF-α Ready-Set-Go Kit (eBioscience, San Diego, CA, USA), respectively, according to the manufacturer’s instructions.

### Flow Cytometry

Peritoneal exudate cells were harvested by washing the peritoneal lavage of mice with 1 ml cold sterile PBS and centrifuging them at 1,500 × *g* for 10 min, at 4°C. FITC-labeled anti-mouse Ly-6C (Gr-1), APC-labeled anti-mouse/human CD11b, and PE-labeled anti-mouse Ly-6G antibodies (all from BioLegend, San Diego, CA, USA) were used for surface antigen staining according to the manufacturer’s instructions. The cells were washed and analyzed in a FACSAria flow cytometer (BD Biosciences). A minimum of 200,000 events was acquired per sample, and the collected data were analyzed in Flow Jo version 10.0 (Tree Star Inc.).

### Statistics

All values are expressed as the mean ± SEM. Data were compared with the two-tailed *t*-test for two groups by using Prism 5.0 (GraphPad Software, Inc.). Significance is showed by *P < 0.05, **P < 0.01, and ***P < 0.001.

## Results

### *N. caninum* Activates the Inflammasome in BMDMs

**Figure 1:**
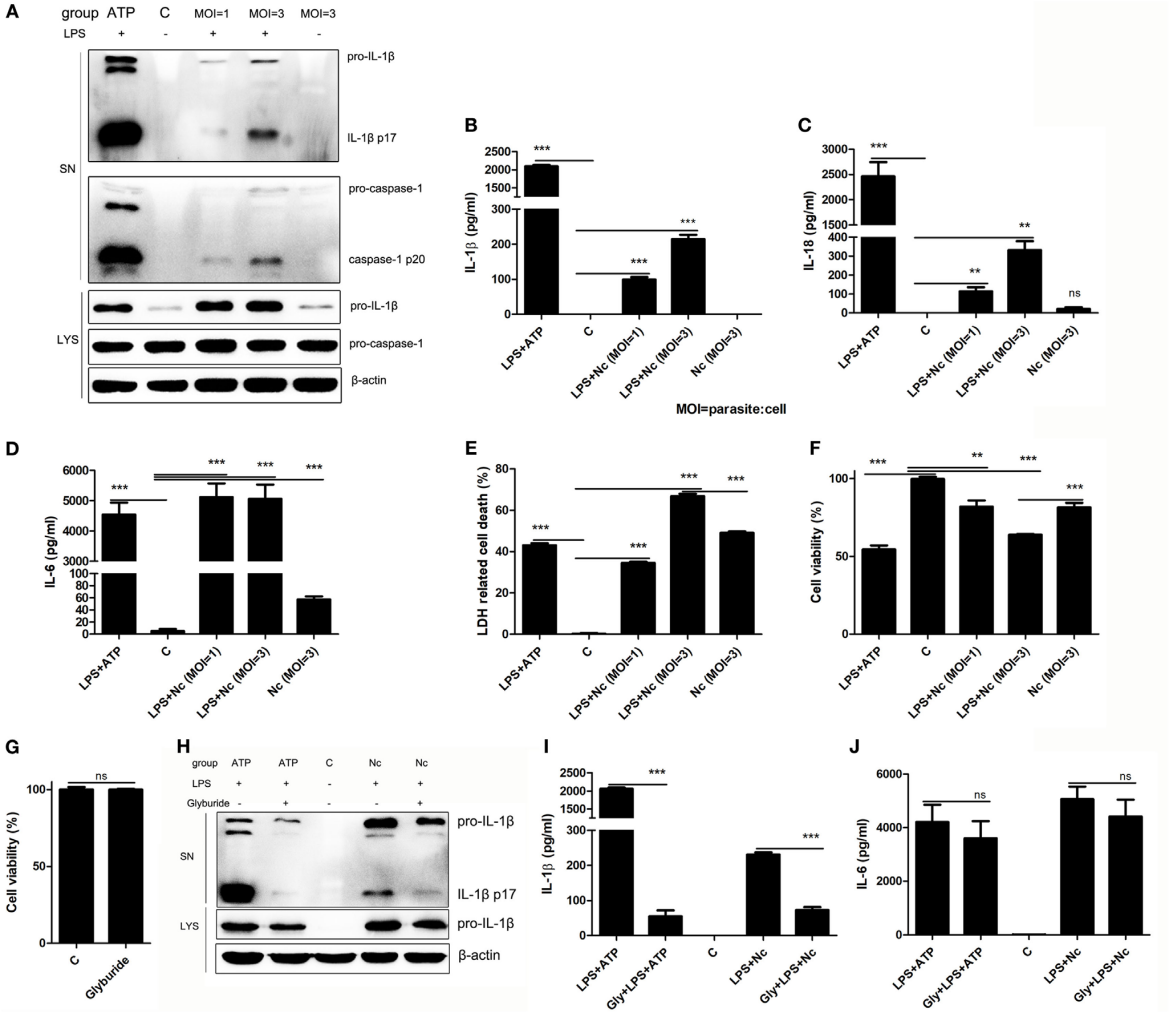
*Neospora caninum* induces IL-1β/IL-18 release, caspase-1 activation, and cell death in bone marrow-derived macrophages (BMDMs). BMDMs were primed with LPS (100 ng/ml) or left unstimulated for 2 h, washed twice with PBS to remove LPS, and subsequently infected with *N. caninum* at MOI = 1 and 3 for 3 h, or with ATP (5 mM, 30 min) as a positive control. **(A)** Cleavage of mature IL-1β (p17) and active caspase-1 (p20) was monitored by Western blotting of supernatants and cell lysates. **(B–D)** IL-1β, IL-18, and IL-6 in supernatants were measured by using ELISA. **(E)** Cell death was monitored by measuring lactate dehydrogenase release in supernatants. **(F)** The viability of infected cells was determined by the Cell Counting Kit (CCK). BMDMs were treated with 50 µM glyburide for 60 min before stimulation and then stimulated with LPS priming plus *N. caninum* (MOI = 3) for 3 h. **(G)** Cell viability of BMDMs treated with 50 µM glyburide for 6 h was determined by the CCK. **(H)** Cleavage of mature IL-1β (p17) was monitored by Western blotting. **(I,J)** IL-1β and IL-6 in supernatants were measured by ELISA. Abbreviations: C, control [with 0.05% DMSO in panels **(H–J)**]; SN, supernatants; LYS, cell lysates; MOI, multiplicity of infection; Gly, glyburide. The data are representative of three independent experiments and are presented as the mean ± SEM (*P < 0.05, **P < 0.01, ***P < 0.001 vs. negative control).

Our previous study showed that *N. caninum* could induce IL-1β release and caspase-1 activation in murine peritoneal macrophages (15). To determine if *N. caninum* also activates the inflammasome in BMDMs, unstimulated and LPS pre-treated BMDMs were infected with *N. caninum* (parasite:cell MOI = 1 or 3) for 3 h. BMDMs with culture medium alone did not produce measurable levels of IL-1β (p17) or active caspase-1 (p20). Like the LPS-pre-treated ATP-stimulated cells, LPS-pre-treated *N. caninum*-infected BMDMs produced measurable levels of IL-1β, and active caspase-1 could be detected after *N. caninum* infection in LPS pre-treated BMDMs; moreover, *N. caninum* alone failed to induce the release of IL-1β or the cleavage of caspase-1 (Figure 1A), which indicate the essential role of LPS pretreatment in *N. caninum*-induced IL-1β release in BMDMs. Like LPS pre-treated ATP-stimulated BMDMs, LPS pre-treated *N. caninum*-infected BMDMs had high levels of both IL-1β and IL-18 in culture supernatants (Figures 1B,C). The production of IL-1β and IL-18, as well as the cleavage of caspase-1 in culture supernatants, depended on the amount of *N. caninum* (Figures 1A–C). The detection of mature IL-1β and IL-18 and cleaved caspase-1 in the *N. caninum*-infected BMDMs indicated inflammasome activation. Meanwhile, high levels of IL-6 mediated by TLRs (27) were detected in LPS pre-treated ATP-stimulated BMDMs, LPS pre-treated *N. caninum*-infected BMDMs, and only *N. caninum*-infected BMDMs at 3 h p.i. (Figure 1D). In addition, ATP treatment caused rapid host cell death, termed pyroptosis (Figures 1E,F). *N. caninum* infection alone (MOI = 3) led to inflammasome-independent cell death of BMDMs (Figures 1E,F) without any detection of caspase-1 cleavage (Figure 1A), while an approximately 15% higher level of cell death was observed in LPS pre-treated *N. caninum*-infected BMDMs (Figures 1E,F), accompanied by caspase-1 cleavage (Figure 1A). These results indicate that the inflammasome was activated in BMDMs by *N. caninum* infection with the release of IL-1β and IL-18 and the cleavage of caspase-1, as well as inflammasome-dependent cell death.

A reduction in intracellular K^+^ is essential for ATP-triggered NLRP3 inflammasome activation (28). To explore whether *N. caninum* also induced NLRP3 inflammasome activation via K^+^ efflux, glyburide, which inhibits the NLRP3 inflammasome by inhibiting K^+^ efflux (29, 30), was added to BMDMs. Treatment with glyburide did not affect the viability of BMDMs (Figure 1G) but did inhibit ATP-induced IL-1β maturation and production (Figures 1H,I). Another group of BMDMs was treated with glyburide, pre-treated with LPS, and infected with *N. caninum* for 3 h, and the maturation and production of IL-1β were significantly reduced (Figures 1H,I). However, glyburide did not greatly impair the production of IL-6 (Figure 1J). Our previous study has shown that glyburide could slightly inhibit the replication of *N. caninum* in Vero cells but not affect its abilities of invasion and viability (15). These data indicate that K^+^ efflux participates in the signals that activate NLRP3 inflammasome in *N. caninum*-infected BMDMs.

### *N. caninum*-Induced Inflammasome Activation in BMDMs Depends on NLRP3, ASC, and Caspase-1/11

**Figure 2:**
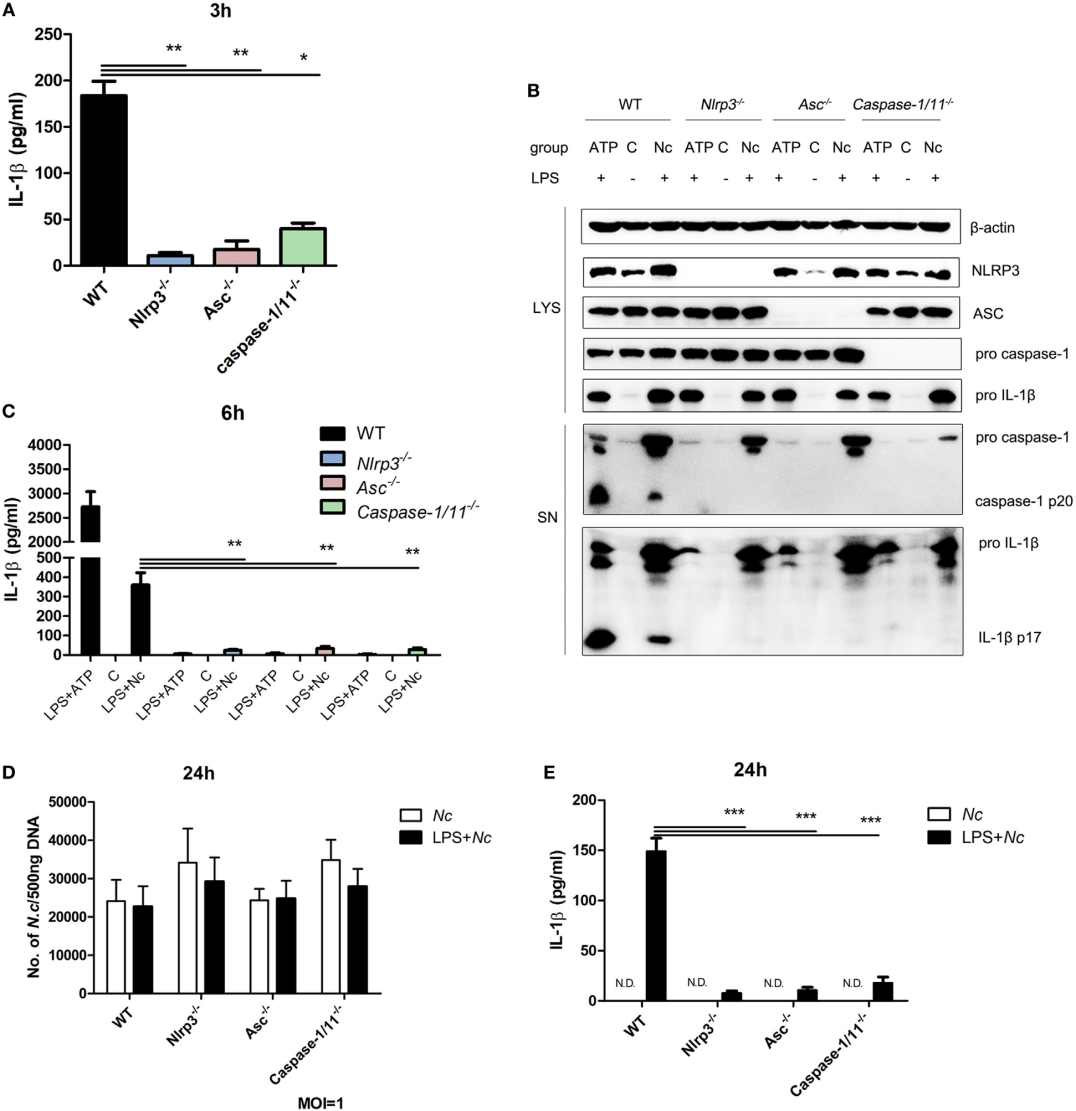
NLRP3, ASC, and caspase-1/11 control inflammasome-mediated IL-1β release in *Neospora caninum* infection. LPS-pre-treated bone marrow-derived macrophages (BMDMs) from wild-type (WT), *Nlrp3^−/−^, Asc^−/−^*, and *Caspase-1/11^−/−^* mice were infected with *N. caninum* at MOI = 3 for 3 or 6 h or stimulated with ATP. **(A)** Production of IL-1β induced by *N. caninum* at 3 h p.i. in supernatants was measured by ELISA. **(B)** Cleavage of mature IL-1β (p17) and active caspase-1 (p20) was detected by Western blotting of supernatants and cell lysates at 6 h p.i. **(C)** IL-1β in supernatants was measured by ELISA at 6 h p.i. BMDMs primed with LPS (100 ng/ml) or left unstimulated were infected with *N. caninum* at MOI = 1 for 24 h. The number of *N. caninum* in infected BMDMs was monitored after 24 h by quantitative PCR **(D)**, and IL-1β in supernatants was measured by ELISA **(E)**. Abbreviations: C, control; SN, supernatants; LYS, cell lysates; Nc, *N. caninum*; N.D., not detected. The data are representative of three independent experiments and are presented as the mean ± SEM (*P < 0.05, **P < 0.01, ***P < 0.001 vs. WT group).

To determine whether the inflammasome components are necessary for IL-1β secretion caused by *N. caninum* infection, BMDMs from mice that lacked NLRP3, ASC, and caspase-1/11 were infected with *N. caninum* (MOI = 3). A greatly reduced amount of active IL-1β in the supernatant of *Nlrp3^−/−^, Asc^−/−^*, and *Caspase-1/11^−/−^* BMDMs infected with *N. caninum* was observed compared with the supernatant of WT BMDMs at 3 h p.i. (Figure 2A). NLRP3 inflammasome-mediated IL-1β release and caspase-1 activation caused by ATP treatment were almost abolished in *Nlrp3^−/−^, Asc^−/−^*, and *Caspase-1/11^−/−^* BMDMs (Figures 2B,C). Similar results were obtained after *N. caninum* infection for 6 h, the cleavage of IL-1β and caspase-1 in culture supernatants was greatly decreased or inhibited in *Nlrp3^−/−^, Asc^−/−^*, and *Caspase-1/11^−/−^* BMDMs (Figures 2B,C). These results indicate the essential roles of NLRP3, ASC, and caspase-1/11 in *N. caninum*-induced inflammasome activation.

To explore if the NLRP3 inflammasome influences *N. caninum* proliferation in BMDMs, BMDMs (both LPS-pre-treated and unstimulated) from WT, *Nlrp3^−/−^, Asc^−/−^*, and *Caspase-1/11^−/−^* mice were infected with *N. caninum* (MOI = 1) for 24 h, and parasite burden was assessed by qPCR with total DNA (500 ng) from infected BMDMs. We observed that the parasite burden in *Nlrp3^−/−^* and *Caspase-1/11^−/−^* BMDMs was higher than that in WT BMDMs. The parasite burden in infected BMDMs (without LPS pretreatment) was slightly higher than that in LPS pre-treated *N. caninum*-infected BMDMs. But no significant differences were observed (Figure 2D). In addition, IL-1β production could only be detected in LPS pre-treated infected BMDMs (Figure 2E). These results imply that *N. caninum* alone could not induce inflammasome activation in BMDMs at 24 p.i. Thus, the activation of inflammasome caused by *N. caninum* in BMDMs needs LPS pretreatment to provide the first signal.

### Roles of NLRP3, ASC, and Caspase-1/11 in Controlling *N. caninum* Proliferation and Host Resistance

**Figure 3:**
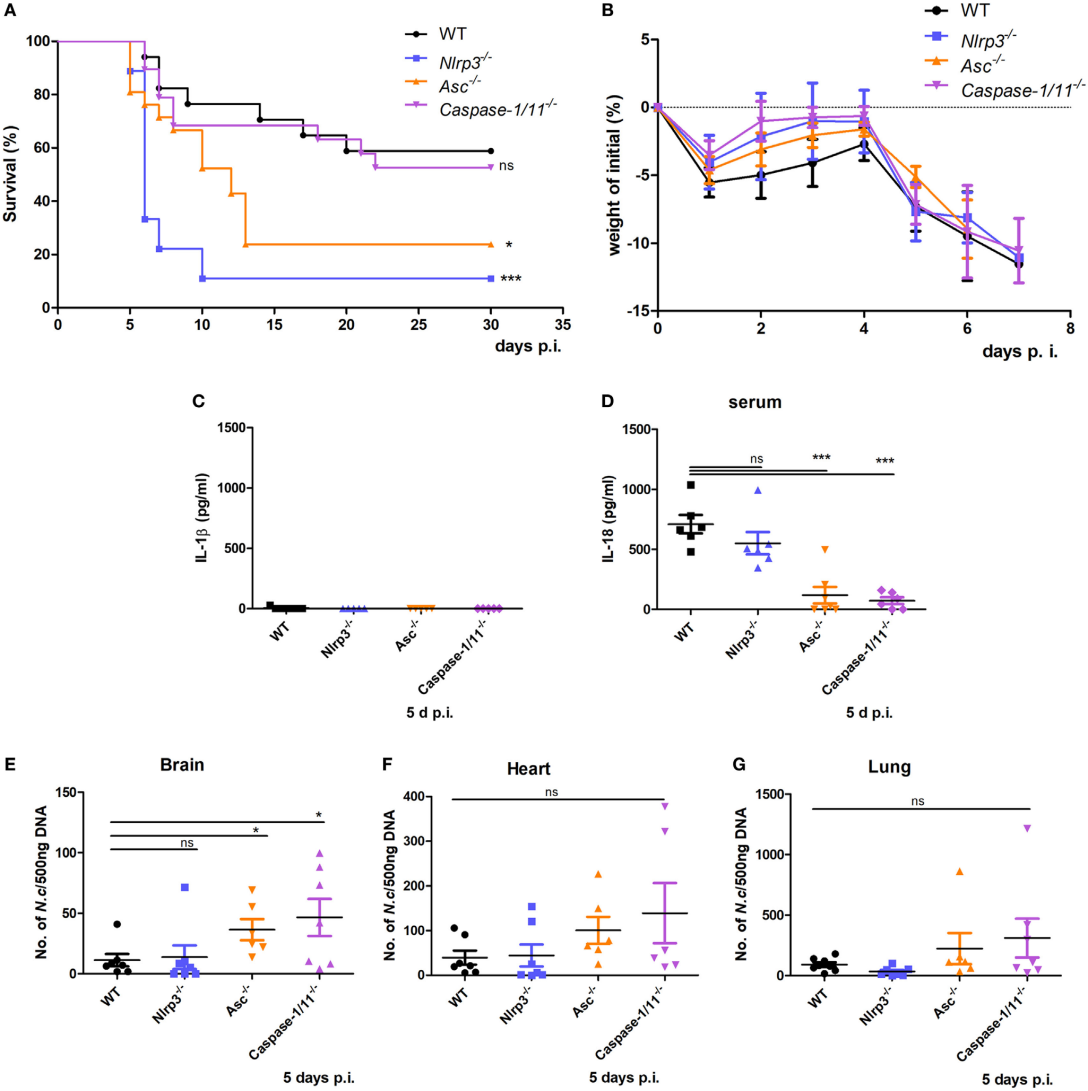
Roles of NLRP3 inflammasome components in host resistance to *Neospora caninum* infection. Wild-type (WT), *Nlrp3^−/−^, Asc^−/−^*, and *Caspase-1/11^−/−^* mice were infected with 2 × 10^7^ of *N. caninum* tachyzoites (intraperitoneal route) and were monitored daily. **(A)** Survival of mice was monitored for 30 days. **(B)** Weight of mice was recorded daily before death occurred. IL-1β **(C)** and IL-18 **(D)** in serum of these mice on day 5 after infection were measured by ELISA. Parasite loads were evaluated in brain **(E)**, heart **(F)**, and lung **(G)** samples by quantitative PCR. Data are shown as the mean ± SEM from three independent experiments **(A,B)** or two independent experiments **(C–G)** (*P < 0.05, **P < 0.01, ***P < 0.001 vs. WT group).

To investigate the role of the NLRP3 inflammasome in murine susceptibility to *N. caninum* infection, WT, *Nlrp3^−/−^, Asc^−/−^*, and *Caspase-1/11^−/−^* mice were infected with 2 × 10^7^ *N. caninum* tachyzoites intraperitoneally and tested for susceptibility to *N. caninum* infection by monitoring survival time, parasite burden and the production of IL-1β and IL-18. In the absence of NLRP3 and ASC, mice were highly susceptible to acute infection, increased mortality was observed (Figure 3A), although no obvious differences in weight loss were found between WT, *Nlrp3^−/−^, Asc^−/−^*, and *Caspase-1/11^−/−^* mice before death occurred (Figure 3B). Interestingly, IL-1β was almost undetectable in serum on day 5 p.i. (Figure 3C). However, IL-18 was significantly higher in WT mice, while IL-18 production was partly reduced in *Nlrp3^−/−^* mice and significantly reduced in *Asc^−/−^* and *Caspase-1/11^−/−^* mice (Figure 3D). In addition, parasite loads in the brain, heart, and lung of *Asc^−/−^* and *Caspase-1/11^−/−^* mice were threefold to fourfold-higher than in WT mice on day 5 p.i. (Figures 3E–G). These results indicate that NLRP3 and ASC play essential roles in protecting the host against *N. caninum* infection and that inflammasome-mediated IL-18 production and elimination of parasites mainly depend on ASC and caspase-1/11, or partially depend on NLRP3 during acute infection.

### NLRP3 Inflammasome Activation Mediates IL-18 and IFN-γ Production in *N. caninum* Infection

**Figure 4:**
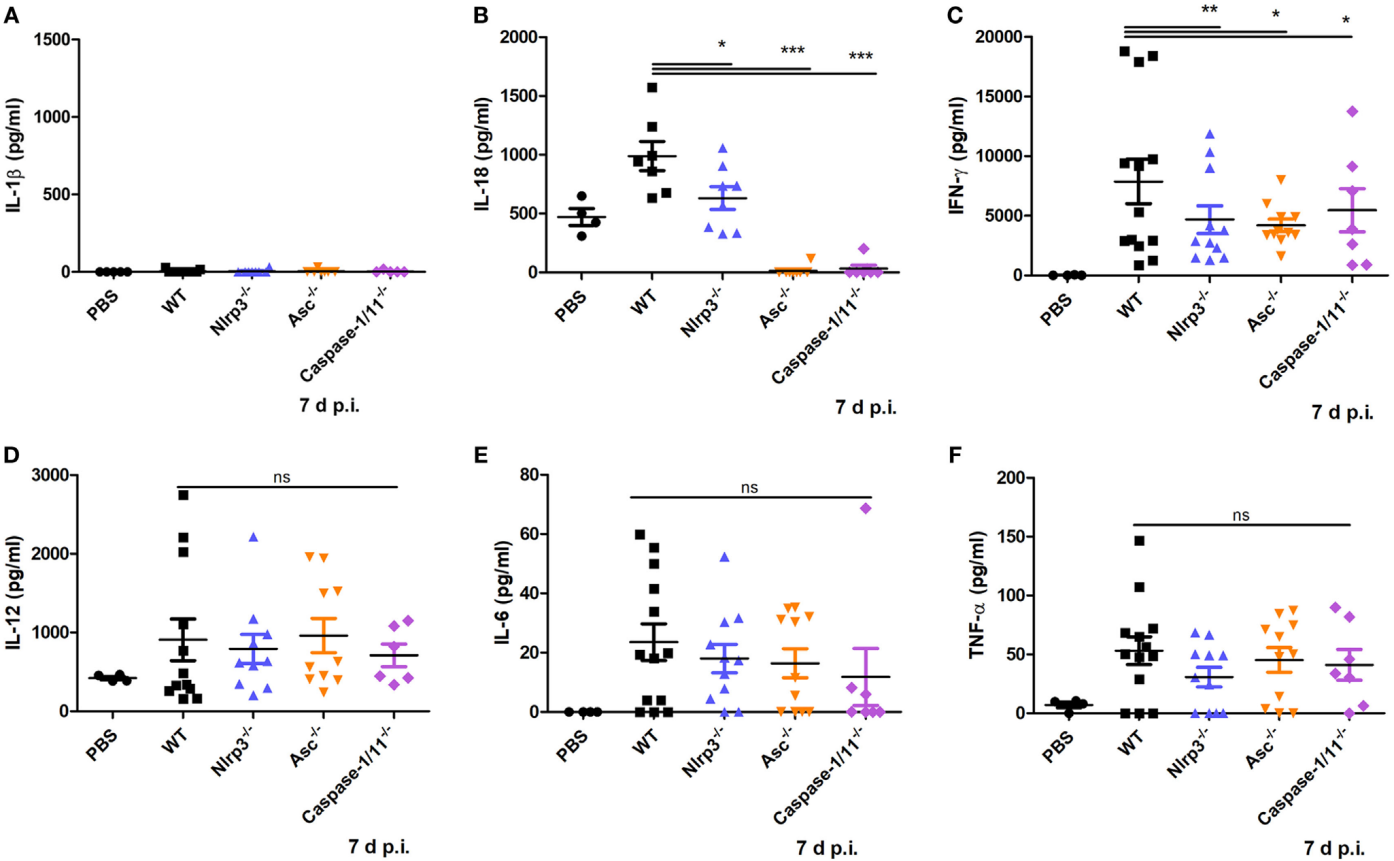
Production of some proinflammatory cytokines at the acute stage of *Neospora caninum* infection. Wild-type (WT), *Nlrp3^−/−^, Asc^−/−^*, and *Caspase-1/11^−/−^* mice were infected with 1 × 10^7^ of *N. caninum* tachyzoites (intraperitoneal route) for 7 days. IL-1β **(A)**, IL-18 **(B)**, IFN-γ **(C)**, IL-12 **(D)**, IL-6 **(E)**, and TNF-α **(F)** were measured by ELISA in serum of infected mice. Data are shown as the mean ± SEM from two independent experiments **(A,B)** or from three independent experiments **(C–F)** (*P < 0.05, **P < 0.01, ***P < 0.001 vs. WT group).

To test if inflammasome activation participates in mediating proinflammatory cytokine production caused by *N. caninum* infection, WT, *Nlrp3^−/−^, Asc^−/−^*, and *Caspase-1/11^−/−^* mice were infected with 1 × 10^7^ *N. caninum* tachyzoites intraperitoneally for 7 days. IL-1β, IL-18, IFN-γ, IL-12, IL-6, and TNF-α in the circulation were measured. IL-1β in serum did not exceed 30 pg/ml (Figure 4A), similar to the result in Figure 3C. High level of IL-18 was detected in WT mice, whereas IL-18 was greatly reduced in the absence of NLRP3, ASC, or caspase-1/11 (Figure 4B). In addition, IFN-γ was significantly decreased in *Nlrp3^−/−^, Asc^−/−^*, and *Caspase-1/11^−/−^* mice compared with WT group (Figure 4C). There were no obvious differences in IL-12, IL-6, or TNF-α between WT, *Nlrp3^−/−^, Asc^−/−^*, and *Caspase-1/11^−/−^* mice (Figures 4D–F). These results indicate that the NLRP3 inflammasome contributes to the secretion of IL-18, as well as the production of IFN-γ.

### NLRP3 Inflammasome Components Participate in the Host Response Against *N. caninum* at the Initial Infection Site

**Figure 5:**
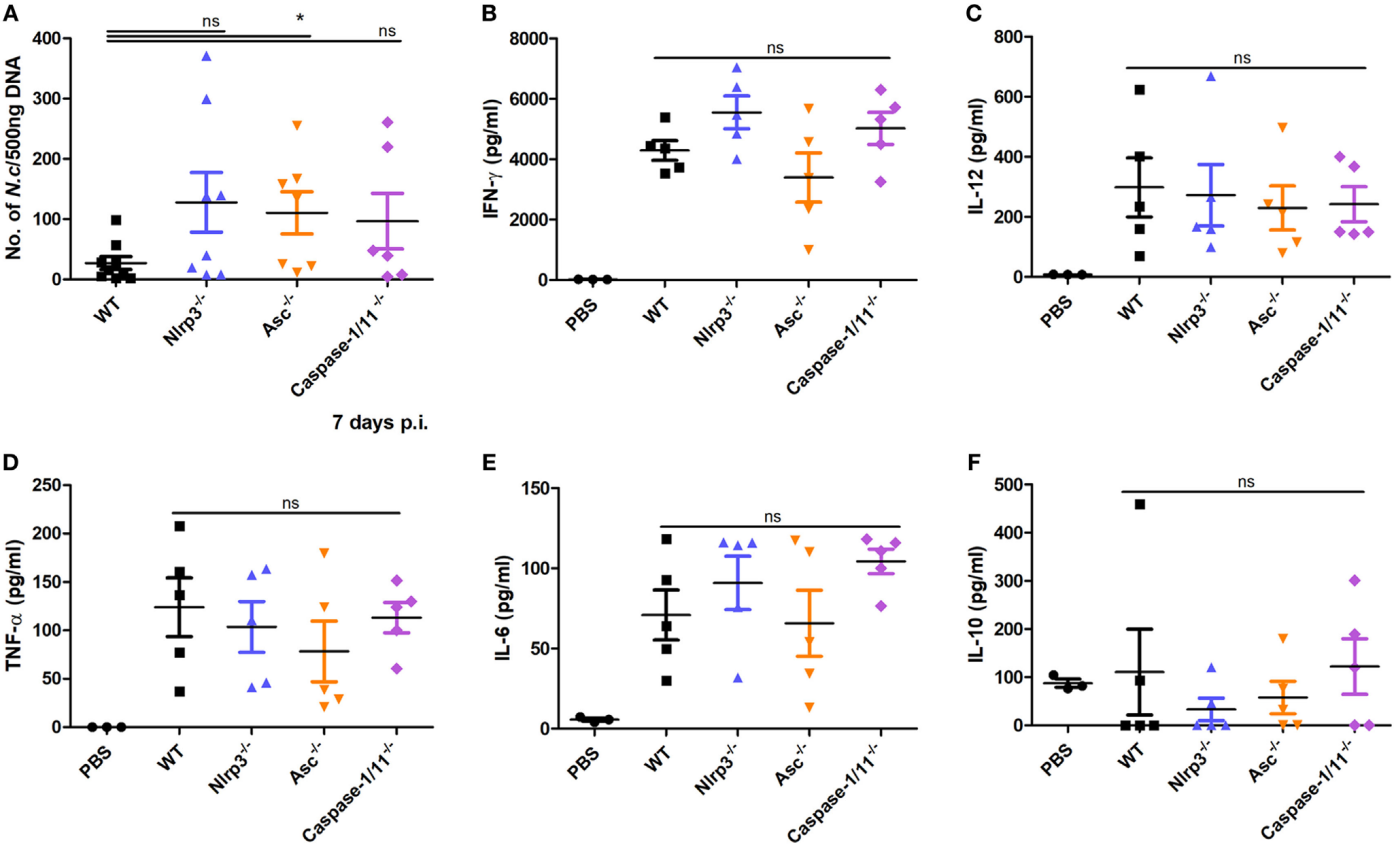
Roles of NLRP3, ASC, and caspase-1/11 in the host response against *Neospora caninum* at the initial infection site. Peritoneal exudate cells and peritoneal exudate fluids from wild-type (WT), *Nlrp3^−/−^, Asc^−/−^*, and *Caspase-1/11^−/−^* mice infected with 1 × 10^7^ of *N. caninum* tachyzoites (intraperitoneal route) for 7 days were collected. **(A)** Parasite loads were measured by quantitative PCR in peritoneal exudate cells. IFN-γ **(B)**, IL-12 **(C)**, TNF-α **(D)**, IL-6 **(E)**, and IL-10 **(F)** were detected by ELISA in peritoneal exudate fluids. Data are shown as the mean ± SEM from two independent experiments (*P < 0.05, **P < 0.01, ***P < 0.001 vs. WT group).

Immune responses at the initial site of infection are important to control parasite replication and dissemination. To explore whether the NLRP3 inflammasome components NLRP3, ASC, and caspase-1/11 mediate the immune responses at the infection site, parasite load in peritoneal exudate cells and cytokines production in peritoneal exudate fluids from mice infected with 1 × 10^7^ *N. caninum* tachyzoites (intraperitoneally) for 7 days were measured. *Nlrp3^−/−^, Asc^−/−^*, and *Caspase-1/11^−/−^* mice had sixfold to eightfold higher parasite loads over WT mice (Figure 5A). No significant differences in IFN-γ (Figure 5B), IL-12 (Figure 5C), TNF-α (Figure 5D), IL-6 (Figure 5E), or IL-10 (Figure 5F) between WT, *Nlrp3^−/−^, Asc^−/−^*, and *Caspase-1/11^−/−^* mice were observed, but *Nlrp3^−/−^* and *Caspase-1/11^−/−^* mice had high productions of IFN-γ and IL-6, and *Asc^−/−^* mice had low productions of these two cytokines (Figures 5B,E). These results demonstrate the essential role of the NLRP3 inflammasome in controlling parasite growth and indicate that the secretions of these cytokines in peritoneal exudate fluids are independent of the NLRP3 inflammasome.

### Roles of NLRP3, ASC, and Caspase-1/11 in the Recruitment of Monocytes/Macrophages and Neutrophils in the Acute Phase of *N. caninum* Infection

**Figure 6:**
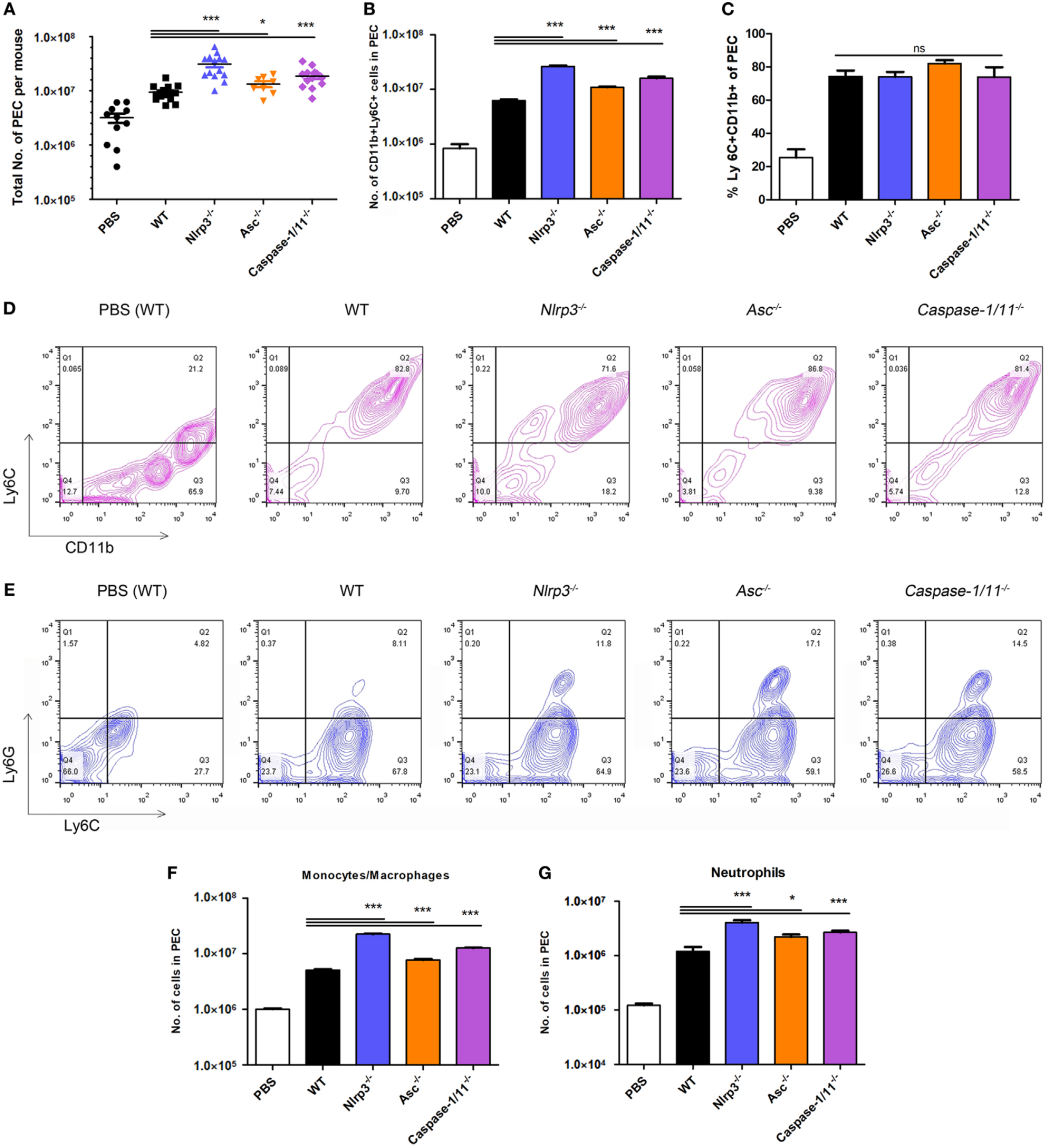
Recruitment of monocytes/macrophages and neutrophils at the acute stage of *Neospora caninum* infection. Wild-type (WT), *Nlrp3^−/−^, Asc^−/−^*, and *Caspase-1/11^−/−^* mice were infected with 1 × 10^7^ of *N. caninum* tachyzoites (intraperitoneal route) for 7 days, and peritoneal exudate cells were collected for flow cytometry analysis. **(A)**. Total numbers of peritoneal exudate cells in each infected mouse were counted. **(B–D)** The influx of monocytes/macrophages and neutrophils (CD11b^+^Ly6C^+^ cells) was evaluated by flow cytometry. Monocytes/macrophages (Ly6C^+^Ly6G^−^ cells) and neutrophils (Ly6C^+^Ly6G^+^ cells) in peritoneal exudate cells were analyzed by flow cytometry **(E)**, and their numbers are shown **(F,G)**. Data are shown as the mean ± SEM from three independent experiments **(A)** or are representative of two independent experiments **(B–G)** (*P < 0.05, **P < 0.01, ***P < 0.001 vs. WT group).

Primary immune cells at the initial site of the infection play important roles in producing proinflammatory cytokines, so we reasoned that the total numbers of peritoneal exudate cells in these groups would be different. We measured the levels of monocytes/macrophages and neutrophils that were recruited into the peritoneal cavity after infection with the parasite. We observed that the absolute numbers of peritoneal exudate cells in *Nlrp3^−/−^, Asc^−/−^*, and *Caspase-1/11^−/−^* mice were significantly higher than those in WT mice (Figure 6A). And *Nlrp3^−/−^, Asc^−/−^*, and *Caspase-1/11^−/−^* mice recruited great numbers of both monocytes/macrophages and neutrophils (CD11b^+^Ly6C^+^ cells) than WT mice (Figure 6B), probably in response to increased numbers of *N. caninum* (Figure 5A). However, the proportions of monocytes/macrophages and neutrophils were similar between these four groups (Figures 6C,D). The influxes of monocytes/macrophages (Ly6C^+^Ly6G^−^ cells) and neutrophils (Ly6C^+^Ly6G^+^ cells) in peritoneal exudate cells were evaluated by flow cytometry (Figure 6E). Significantly higher numbers of monocytes/macrophages (Figure 6F), and considerably more neutrophils (Figure 6G) were recruited into the peritoneal cavity of *Nlrp3^−/−^, Asc^−/−^*, and *Caspase-1/11^−/−^* mice compared to WT mice (Figures 6F,G). These results may suggest that high production of IFN-γ and IL-6 in peritoneal exudate fluids of *Nlrp3^−/−^* and *Caspase-1/11^−/−^* mice (Figures 5B,E) are related to the high numbers of monocytes/macrophages and neutrophils (Figures 6F,G) and may suggest that NLRP3, ASC, and caspase-1/11 are not essential for the recruitment of monocytes/macrophages and neutrophils.

## Discussion

During infection, immune cells first recognize invasive microbes and initiate an immediate innate immune response. This immune response is important in eliminating pathogens, and it can mediate appropriate adaptive immune response to promote and strengthen the elimination of invasive microbes, as well as build immunological memory to protect against reinfection (31). Understanding of the immune responses against *N. caninum* is limited. We have previously demonstrated that the NLRP3 inflammasome can be activated in *N. caninum* infection *in vitro* (15). In this study, we focused on the role of the NLRP3 inflammasome in mediating the immune response against *N. caninum* at the acute stage of infection.

NLRP3 inflammasome-mediated IL-1β/IL-18 release depends on its structural domains: the sensor NLRP3, the adaptor molecule ASC, and caspase-1 (16). The activation of the NLRP3 inflammasome in macrophages requires two signals. The first signal is provided by NF-κB activation, which causes the upregulation of NLRP3, pro-IL-1β, and pro-IL-18. The second signal is provided by various DAMPs or PAMPs (32, 33). Activation of the NLRP3 inflammasome in response to diverse stimuli has been proposed to be triggered by multiple cellular signals: K^+^ efflux, Ca^2+^ signaling, mitochondrial dysfunction, and lysosomal rupture (34, 35). In this study, the release of IL-1β and IL-18 induced by *N. caninum* infection in BMDMs required the presence of LPS pretreatment, because *N. caninum* alone failed to induce IL-1β release at 3 or 24 h p.i. However, in our previous study, *N. caninum* alone triggered the maturation of IL-1β in peritoneal macrophages (15). This difference may be related to the inherent differences between BMDMs and peritoneal macrophages (36). Meanwhile, K^+^ efflux in *N. caninum*-infected BMDMs promoted the activation of NLRP3 inflammasome, whether other signals also contribute to inflammasome activation in *N. caninum*-infected BMDMs needs further exploration. Increasing numbers of reports suggest that NLRP3 inflammasome activation facilitates the restriction of microbial replication (37–39). In some studies of protozoan infections, without the presence of NLRP3, ASC, and caspase-1/11 (or caspase-1) in mice, the clearance of *Leishmania* (40), *T. gondii* (20), *Trypanosoma cruzi* (41, 42) is impaired, and mice are more susceptible to invasive pathogens. It is interesting to note that administration of inflammasome activation has provided people with potential means for the treatment and prevention of infection. Extracellular ATP treatment could restrain the proliferation of *T. gondii* in macrophages or small intestinal epithelial cells by inducing NLRP3 inflammasome activation (43, 44). Meanwhile, treatment with IL-18 can serve to control leishmaniasis by inducing Th1 response and improve host resistant to reinfection by inducing and/or activating memory cells against *Leishmania major* infection (45). In addition, involvement of NLRP3 inflammasome activator in adjuvants can effectively promote the adaptive immune response to vaccination (23, 46). Our data reveal that maturation of IL-1β and cleavage of caspase-1 were almost abolished in *N. caninum*-infected BMDMs from *Nlrp3^−/−^, Asc^−/−^*, and *Caspase-1/11^−/−^* mice, and *in vivo* experiments on *N. caninum* infection show that NLRP3 inflammasome components were essential to the clearance of *N. caninum*, the survival of mice, and induction of Th1 response.

There is an interesting relationship between mouse survival and *N. caninum* burden in tissues. In some cases, parasite burden does not contribute to mouse death during *N. caninum* infection. For example, the survival rate of *N. caninum*-infected *CCR5^−/−^* mice was significantly decreased, but no difference was found in the parasite burden of tissues compared with WT mice (47). *N. caninum*-infected *Nod2^−/−^* mice showed increased parasite burden in tissues, but the survival rate of *Nod2^−/−^* mice was also higher than that of WT mice (10). In our study, *N. caninum*-infected *Nlrp3^−/−^* mice showed high mortality but no difference in parasite burden when compared to WT mice. It seems that there is no absolute causal relationship between mouse survival and *N. caninum* burden in tissues. Some studies showed that *Caspase-1/11^−/−^, Caspase-1^−/−^*, and *Caspase-11^−/−^* mice showed differences in survival compared to WT mice. A study of *T. gondii*-infected *Caspase-1/11^−/−^* mice showed decreased survival compared with WT mice (20), but another study found that the survival rate of *Caspase-11^−/−^* mice infected with *T. gondii* was greatly increased (48). Another study found that *Caspase-1^−/−^* mice were significantly more susceptible to infection with *S. typhimurium* than mice lacking both caspase-1 and caspase-11 (49). After infection with LPS, the survival rates of *Caspase-1^−/−^* and *Caspase-11^−/−^* mice were significantly lower than that of mice lacking both caspase-1 and caspase-11 (50, 51). In our study, *N. caninum*-infected *Caspase-1/11^−/−^* mice showed no difference in survival, but produced greatly decreased IL-18 compared with that in WT mice. We infer that caspase-11 may affect the susceptibility of caspase-1 to *N. caninum* infection.

During *N. caninum* infection, protective immune responses are typically dependent on the Th1 response, mainly mediated by the secretion of IFN-γ and IL-12, and these cytokines play important roles in the clearance of the parasite and host resistance (9, 52). In the absence of IFN-γ, *N. caninum*-infected BALB/c mice died quickly, failed to induce T-cell proliferation and high level of NO production. Moreover, the increase in MHC class II expression on macrophages was impaired (52). Administering IFN-γ-expressing CD8^+^ T cells to *N. caninum*-infected mice resulted in a lower parasitic burden than mice receiving IFN-γ-deficient CD8^+^ T cells (53). Various immune cells, such as NK, NK T, and TCRγδ^+^ cells, CD4^+^ and CD8^+^TCRβ^+^ lymphocytes account for the production of IFN-γ, and interferon-inducible GTPases and nitric oxide synthase are upregulated with the increase in IFN-γ production. All of these effects promote host control of intracellular parasite growth (54). In our study, WT mice infected with *N. caninum* produced a high level of IFN-γ in serum, while the IFN-γ production of *Nlrp3^−/−^, Asc^−/−^*, and *Caspase-1/11^−/−^* mice was significantly impaired, accompanied by severe defects in IL-18 production. This phenomenon is similar to the decreased IL-18 in serum of *Nlrp3^−/−^, Asc^−/−^*, and *Caspase-1/11^−/−^* mice infected with *T. gondii* (20). In addition, both *T. gondii* and *N. caninum* failed to induce the production of IL-1β *in vivo*, but the detailed mechanism remains unknown.

IL-18 exerts important effects on the initiation of the adaptive Th1 cellular responses to infections by inducing IFN-γ. IL-18 is one of the best-characterized inflammasome-dependent cytokines. It is cleaved by caspase-1 from its inactive intracellular precursor pro-IL-18 (55). IL-18 was initially regarded as interferon-γ-inducing factor and has a synergistic effect with IL-12. It markedly induces IFN-γ production in Th1 cells, and this ability does not depend on IL-12. In addition, IL-18 promotes the proliferation of T cells (55, 56). In this study, IFN-γ in serum of *Nlrp3^−/−^, Asc^−/−^*, and *Caspase-1/11^−/−^* mice infected with *N. caninum* was greatly decreased with impaired production of IL-18 compared with WT mice, but IL-12 in these mice was not altered. This result indicates that IL-18 production induced by *N. caninum* infection participates in the induction of IFN-γ. More evidence shows the important role of IL-18 in promoting Th1 responses and shaping adaptive immunity (24). IL-18 is produced by various types of cells, such as Kupffer cells, macrophages, T cells, B cells, and dendritic cells. IL-18 signaling depends on a receptor, IL-18R, which in general is poorly expressed on naïve T cells. With priming, IL-18R is upregulated and binds IL-18, leading to enhancement of Th1 polarization in activated T lymphocytes (57). This enhancement of the activity of IL-18 in Th1 response does play a key role in microbial clearance. In *T. gondii*-infected *Il18^−/−^* and *Il18r^−/−^* mice, parasite replication was increased, and murine survival was impaired (20). *Il18r1^−/−^* mice were highly susceptible to *T. cruzi* infection and had high parasite load (58). *Leishmania* infection caused higher parasite burden and significantly increased lesion size in *Il18^−/−^* mice (59). IL-18 also plays a protective role in the clearance of viruses (60, 61). In our study, IL-18 production seemed to correlate with the elimination of the parasite. When compared with WT mice, IL-18 production was significantly reduced in *Asc^−/−^* and *Caspase-1/11^−/−^* mice, while the parasite burden in the brain was greatly increased compared to WT mice. Although *Nlrp3^−/−^* mice died quickly, WT and *Nlrp3^−/−^* mice showed no great difference in IL-18 production, and these two kinds of mice showed no difference in parasite burden in tissues. These data indicate that production of IL-18 can promote the elimination of *N. caninum* in tissues.

The recruitment of inflammatory monocytes to sites of infection is essential to control parasite growth and dissemination in *T. gondii* and *N. caninum* infections (62, 63). Innate immune responses at the initial infection site are critical for protection against these infections, and cytokines such as IFN-γ (52, 64) and IL-12 (65) help to control these parasites. Innate immune cells that produce and respond to these cytokines include neutrophils, macrophages, DCs, and NK cells (66). Excreted and secreted antigens of *N. caninum* triggered monocytes/macrophages migration to the site of infection in a CCR5-dependent manner (63), and the majority of innate immune cells at the initial infection site of *N. caninum* are monocytes/macrophages and neutrophils (47). We conclude that these two kinds of cells play important roles in the response against *N. caninum*. Mice with the depletion of macrophages have increased susceptibility to *N. caninum* infection (67). In our study at the initial infection site of *Nlrp3^−/−^, Asc^−/−^*, and *Caspase-1/11^−/−^* mice infected with *N. caninum*, the numbers of both monocytes/macrophages and neutrophils were increased, but they failed to limit the *N. caninum* replication, and cytokines IFN-γ, IL-12, TNF-α, IL-6, and IL-10 were not altered when compared with WT mice. These results demonstrate the essential role of the NLRP3 inflammasome in controlling parasite growth. We speculate that pyroptosis may have contributed to the clearance of *N. caninum*. It is worth noting that both lymphocytes and some innate immune cells, such as monocytes/macrophages and neutrophils, can secrete IFN-γ. However, production of IFN-γ by lymphocyte needs the induction of inflammasome-triggered IL-18 (24). Therefore, in our study, the production of IFN-γ was reduced in serum but was not altered at the initial infection site of *Nlrp3^−/−^, Asc^−/−^*, and *Caspase-1/11^−/−^* mice when compared with WT mice during *N. caninum* infection.

Inflammasome-triggered cell death is called pyroptosis, which is an inflammatory form of cell death that plays a key role in controlling invading pathogenic bacteria and microbial infections (68). Upon activation, inflammasome-mediated caspases cleave GSDMD into an N-terminal portion (GSDMD-NT) and a C-terminal portion. GSDMD-NT can oligomerize in the plasma membrane to form pores, thereby causing pyroptosis (69, 70). In our study, *N. caninum* alone caused a high percentage of cell death in BMDMs without the cleavage of caspase-1, while *N. caninum* triggered a higher proportion of cell death with the cleavage of caspase-1 in LPS pre-treated BMDMs. We inferred that the increased cell death in LPS pre-treated BMDMs may be attributed to caspase-1-mediated cell death, termed pyroptosis. Because *N. caninum* alone can cause a high proportion of cell death in BMDMs, it is hard to evaluate pyroptosis in *Nlrp3^−/−^, Asc^−/−^*, and *Caspase-1/11^−/−^N. caninum*-infected BMDMs accurately. *T. gondii* induces pyroptosis in rat macrophages by activating the NLRP1 inflammasome, and this cell death promotes the killing of *T. gondii* (21, 71), but *T. gondii* fails to cause pyroptosis in mice BMDMs (20). Their different abilities to induce pyroptosis in mouse BMDMs may be related to the differences between these two closely parasites (12). Not only does pyroptosis promote bacteria clearances (31) but GSDMD-NT can directly kill bacteria *in vitro* (70). In addition, bacteria released from pyroptotic macrophages can be killed by neutrophils or perhaps reactive oxygen species-producing macrophages (72, 73). In our *in vivo* study with increased numbers of monocytes/macrophages and neutrophils, the parasite loads in peritoneal exudate cells of *Nlrp3^−/−^, Asc^−/−^*, and *Caspase-1/11^−/−^* mice were higher than WT mice, indicating that NLRP3 inflammasome-mediated pyroptosis or GSDMD may play an important role in controlling *N. caninum* replication. More work is needed to confirm the role of pyroptosis in *N. caninum* infection in GSDMD-deficient mice.

In the work described here, we examined the role of NLRP3, ASC, and caspase-1/11 in mediating host immune defense against *N. caninum* in the acute periods of infection. Our data suggest that in the absence of NLRP3, ASC, or caspase-1/11, the parasite burden and mortality rate were higher. NLRP3 inflammasome was essential to mediate the Th1 response by enhancing IL-18 and IFN-γ production during the acute stage of *N. caninum* infection. Understanding the role of NLRP3 inflammasome activation in *N. caninum* infection may contribute new insights into the development of therapeutic options or vaccine strategies to combat this pathogen.

## Ethics Statement

This study was carried out in accordance with the recommendations of guidelines from the Animal Welfare and Research Ethics Committee. The protocol was approved by Jilin University.

## Author Contributions

Conceived and designed the experiments: XW, PG and XCZ. Performed the experiments: XW, XZ, SL, XL, CZ, and QY. Analyzed the data and wrote the paper: XW, ZW, QL, and JL. Supplied reagents: YY and ZY.

## Conflict of Interest Statement

The authors declare that the research was conducted in the absence of any commercial or financial relationships that could be construed as a potential conflict of interest.
